# Development of Viscoplastic Constitutive Model Considering Heating Rate Effect on Grain Size and Phase Evolution in Hot Deformation

**DOI:** 10.3390/ma18143251

**Published:** 2025-07-10

**Authors:** Zheng Gao, Shengyu Liu, Jiatian Lin, Zhihan Wang, Dechong Li, Kailun Zheng

**Affiliations:** School of Mechanical Engineering, Dalian University of Technology, Dalian 116024, China

**Keywords:** optimized viscoplastic constitutive model, heating rate, current-assisted hot stamping, finite element analysis, microstructure

## Abstract

The heating rates and forming temperatures during the hot forming process of titanium alloys cause significant differences in phase transformation, grain size, and dislocation evolution. The formability and service performance of titanium alloy formed components are affected by these factors. This study investigated the hot flow behaviors of Ti-6Al-4V at temperatures ranging from 800 to 900 °C and heating rates ranging from 0.1 to 10 °C/s. These were tested via Gleeble hot tensile experiments, and the grain size and phase evolution were quantitatively characterized via EBSD and XRD. The results suggest that a higher heating rate decreases the β-phase transformation and dislocation density and inhibits grain coarsening, leading to better formability. The heating rate was introduced into the viscoplastic constitutive model for the first time to achieve accurate predictions of the microstructure and hot flow behavior under different heating rates. The prediction accuracy of the hot flow behavior and phase volume fraction reaches 92.93% and 94.97%. The current-assisted hot stamping experiments and finite element (FE) simulations of Ti-6Al-4V irregular cross-section components were carried out at temperatures of 800 and 900 °C and at heating rates of 1 and 3 °C/s. The results show that the rapidly heated formed components exhibit better thickness uniformity and yield strength. The FE simulation guided by the optimized constitutive model has achieved a 96.96% and 92.76% prediction accuracy for the thickness distribution and β-phase volume fraction, respectively.

## 1. Introduction

Ti-6Al-4V is an α + β-type titanium alloy characterized by high specific strength and superior thermal stability, meeting the requirements for high-temperature resistant, lightweight, and thin-walled components for advanced equipment such as aircraft engine blades and rocket nozzles [1,2]. However, titanium alloys are difficult to form at room temperature due to their poor plastic deformation capacity, high deformation resistance, and severe rebound. Therefore, it is necessary to increase the forming temperature to improve the forming properties of titanium alloys [3,4].

In recent years, in order to overcome the weak formability at room temperature of titanium alloy, various hot forming processes have been developed, such as superplastic forming [5], hot stamping forming [6], hot gas pressure forming [7], and isothermal hot stamping techniques [8]. Liu et al. [9] proposed a superplastic forming process of Ti-6Al-4V combined with deep drawing at 800–900 °C. The results showed that the thickness decrease at the outer corner of the superplastic-formed components reached 54%, and cracks and oxidation occurred on the surface of the sheet. Wang et al. [10] investigated the hot gas pressure forming process of Ti-55 tubular components at 750–950 °C. But the die was heated by induction heating, which had a long heating waiting time and low forming efficiency. Qu et al. [11] investigated the effect of forming parameters such as pressure, temperature, initial tube diameter, and weld position on the hot gas pressure forming process of TC2 tubes within the range of 750–900 °C. Although the forming time of the component was shortened to 30 min, the process parameters were complex and the forming stability was relatively low. Furthermore, in the above-mentioned process, the sheets and dies are long-term exposed to high temperatures, which leads to high manufacturing and maintenance costs for the die and causes micro-grain coarsening of the sheet. All these will affect the forming performance of the alloy [12,13]. Kopec et al. [14] proposed a cold-die hot stamping process for Ti-6Al-4V. Hot stamping experiments indicated that obvious cracks appeared on the surface of the formed component at 900 °C. This was attributed to the cooling process during transfer and stamping, which led to martensitic transformation and the decline in formability. Maeno et al. [15] studied the resistance-heating hot stamping process of Ti-6Al-4V sheet concave-bottom U-shaped components and found that current-assisted heating can decrease heating time and stamping time. Ozturk et al. [16] investigated the applicability of the current-assisted heating process to the hot formability of Ti-6Al-4V. And the results showed that current-assisted heating to 700 °C did not affect the hardness and microscopic grains of the alloy, and above 600 °C it could decrease springback. Therefore, the current-assisted stamping process for rapid heating of the sheet decreases the cost of die manufacturing, and decreases heat drop during the transfer process. This is a solution for the efficient stamping of titanium alloy components.

During the current-assisted heating process, the heating rate is an important parameter. Studying the heating rate’s effect on flow stress and microstructure evolution in Ti-6Al-4V is of crucial significance. Ismail et al. [17] investigated the phase transformation kinetics of Ti-6Al-4V at heating rates ranging from 0.03 to 200 °C/s. They analyzed the changes in the average lattice parameters and full width at half maximum of the β phase at different heating rates and concluded that the dissolution of the α phase was controlled by solute element diffusion. Kent et al. [18] investigated the aging response of Ti-6Cr-5Mo-5V-4Al under different temperatures and heating rates. The results indicated that a slower heating rate (5 °C/min) increased the nucleation number of α phase and resulted in finer and more uniformly distributed precipitates. The high heating rate (~100 °C/min) increases the nucleation of the α phase at the α/β interface, resulting in coarser and unevenly distributed precipitates which also coarsen more rapidly. Wang et al. [19] studied the influence of heating parameters on the ductility and post-forming strength of Ti-6Al-4V based on hot stamping engineering problem. The results show that rapid heating leads to the insufficient diffusion of V elements, forming a non-equilibrium microstructure, decreasing phase transformation, and improving the formability of the alloy. After deformation, nano-scale martensite with high dislocation density is formed inside the β phase, which is conducive to maintaining the post-forming strength. Li et al. [20] investigated the influence of rapid heating on the hot forming and microstructure of Ti-6Al-4V. The results indicated that a low heating rate promoted the α- to β-phase transformation and increased the ductility below 900 °C. In addition, a high heating rate improved formability at 950 °C. The model considering phase transformation was established to predict the evolution of phase volume fraction. Xiao et al. [21] optimized the Arrhenius constitutive equation and applied it to the FE model to simulate hot forming process of Ti-6Al-4V thin-walled components, which is helpful for formulating special process routes. Li et al. [22] studied the effects of heating rate on the phase transformation and strength of TA32 and embedded the viscoplastic model of TA32 into the FE model through the VUMAT subroutine to realize the simulation of the forming process. Therefore, current research on the heating rate of Ti-6Al-4V hot stamping mainly focuses on the microstructure evolution of alloys and the mechanical strength of the formed components. Moreover, existing material models of Ti-6Al-4V lack consideration of the heating rate. Integrating the heating rate-related model of microstructure evolution into the viscoplastic constitutive model and embedding it to the FE model to simulate the current-assisted hot stamping process is necessary for engineering practice.

In this study, the effect of heating rate on the hot flow behaviors and microstructure evolution of Ti-6Al-4V is investigated through Gleeble hot tensile tests. The heating rate is introduced into the viscoplastic constitutive model, and this model is embedded into the FE simulation subroutine to predict the stamping process of titanium alloy irregular cross-section components. The current-assisted hot stamping experiment was carried out using a current-assisted hot stamping device to verify the validity of the model.

## 2. Materials and Methods

### 2.1. High Temperature Tensile Tests

Ti-6Al-4V annealed-state sheets with a thickness of 1.5 mm were used for hot uniaxial tensile tests, and these were provided by Baoti Group Ltd., Baoji, China. The main chemical composition (wt%) is listed in Table 1 [20].

**Figure 1:**
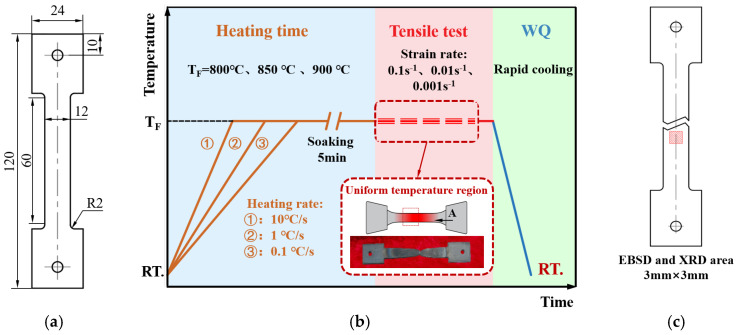
Hot uniaxial tensile tests. (**a**) Size of tensile specimen; (**b**) tensile test process; (**c**) microstructure samples selected position.

The specimens were cut along the rolling direction (RD) of the sheets, and the tensile tests were carried out using a Gleeble 3800, which is from Dynamic Systems Inc., Poughkeepsie, NY, USA and accurately controlled the heating rate and temperature. The tensile tests were carried out following Figure 1. The tensile temperatures ranged from 800 to 900 °C, the heating rates ranged from 0.1 to 10 °C/s, and the strain rates ranged from 0.001 to 0.1 s^−1^. The fracture specimens were water quenched immediately after soaking for 1 s to maintain the microstructure caused by the high temperatures.

### 2.2. Current-Assisted Hot Stamping Experiments

**Figure 2:**
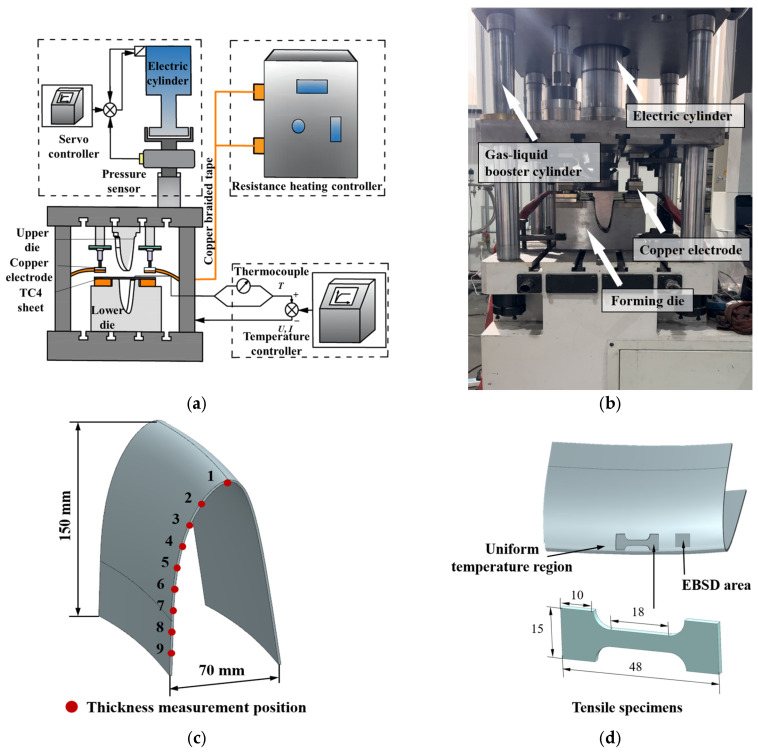
(**a**) Schematic diagram of current-assisted hot stamping device; (**b**) current-assisted hot stamping device; (**c**) irregular cross-section component and thickness measurement positions; (**d**) cutting position of microstructure samples and tensile specimens.

In addition, current-assisted hot stamping experiments with controllable heating rates were conducted to test the effect of different heating rates on the actual stamping process of Ti-6Al-4V components. The experimental device is shown in Figure 2a. The forming die is mounted on a double-action press, the Ti-6Al-4V sheet is placed in the middle position of the copper electrode, the crimping cylinder presses the copper electrode to make it contact with the sheet, and, based on the principle of resistive heating, different amounts of currents are passed into the sheet to achieve different heating rates. The experimental temperature is monitored and controlled via thermocouples by a heating controller. The actual stamping device shown in Figure 2b was constructed and Ti-6Al-4V sheet were selected for the experiments with a thickness of 1.5 mm. The sheets were heated to 800, 850, and 900 °C at a heating rate of 1 and 3 °C/s. Subsequently, the electric cylinder of the press drove the upper die to move down and made the clamping die quickly complete the stamping. According to the method shown in Figure 2c, the thickness of the irregular cross-section components was measured at different positions to investigate the effect of the heating parameters on the thickness uniformity of the irregular cross-section components, and to ensure the validity of the measurement results the thickness measurement at the same position was measured three times to obtain the average data. Using the method shown in Figure 2d, tensile specimens and electron backscatter diffraction (EBSD) microstructure samples were cut in the uniform temperature region of the irregular cross-section components to test the mechanical properties and microstructure.

### 2.3. Microstructure Characterization

The microstructure samples of the tensile and irregular cross-section components were observed, and quantitative statistical methods were performed to obtain the phase volume fraction and grain size evolution using X-ray diffraction (XRD) (using an XRD-6000 X-ray diffractometer, which is from Shimadzu Corporation, Kyoto, Japan) and EBSD (using an SU5000 field emission scanning electron microscope, which is from Hitachi High-Tech Corporation, Tokyo, Japan). The microstructure samples were processed by grinding and electrolytic polishing for the quantitative observation of the microstructure. The electropolishing was performed using a solution of 10% HClO_4_ + 90% C_2_H_5_OH with a current of 1 A and a voltage of 20 V for a duration of 20 s with an EBSD scanning step of 0.4 μm. The obtained electron microscopy images were processed and analyzed using AztecCrystal 2.1.2 and TSL OIM Analysis 6 software which revealed the effect of heating rate on the microstructure evolution.

## 3. Results and Discussion

### 3.1. Characterization of the Ti-6Al-4V Initial Materials

**Figure 3:**
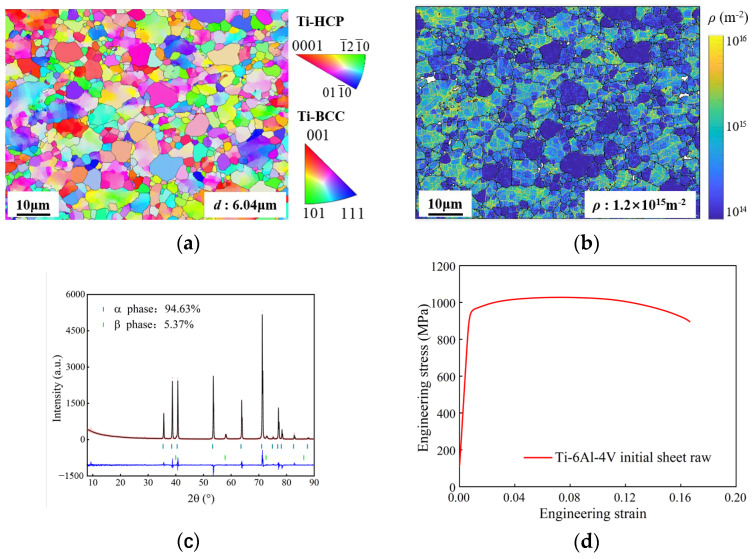
Microstructure and room temperature strength of Ti-6Al-4V initial sheet. (**a**) Inverse pole figure (IPF) map; (**b**) geometrically necessary dislocation (GND) map; (**c**) XRD map of phase volume fraction; (**d**) engineering stress–strain curve at room temperature.

The microstructure of an initial sheet is shown in Figure 3. The grain size is 6.04 μm, the α-phase volume fraction is 94.63%, the β-phase volume fraction is 5.37%, and the dislocation density is 1.2 × 10^15^ m^−2^. The yield strength is 895 MPa and the ultimate tensile strength is 1027 MPa.

### 3.2. Effect of Heating Rate and Temperature on Hot Deformation

**Figure 4:**
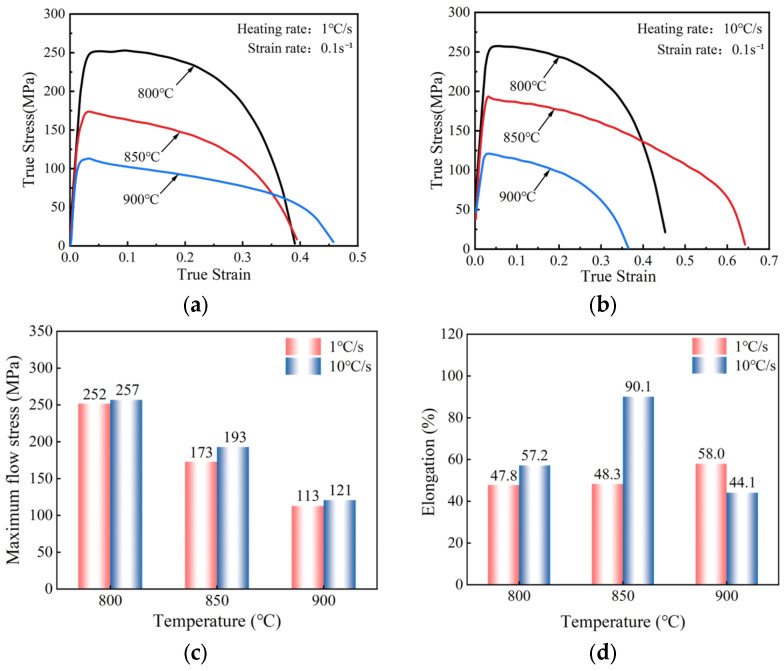
Hot tensile test results of Ti-6Al-4V under different tensile temperatures and heating rates. (**a**) 1 °C/s-0.1 s^−1^; (b) 10 °C/s-0.1 s^−1^; (**c**) the maximum flow stress; (**d**) the elongation of the titanium alloy.

Figure 4 presents the hot tensile results under different heating rates and temperatures. As shown in Figure 4a,b, the maximum flow stress of the Ti-6Al-4V decreases and the elongation increases when increasing the experimental temperature. As the heating rate increases, the maximum flow stress and elongation increase. Figure 4c shows that the maximum flow stress increased from 173 to 193 MPa when the heating rate increased from 1 to 10 °C/s at 850 °C. The maximum flow stress increased by 11.56% with the increase in heating rate. As the experimental temperature increases from 800 to 900 °C, the maximum flow stress decreases from 257 to 121 MPa, which is 52.92% lower. Figure 4d shows the elongation of the material. When the heating rate at 850 °C increased from 1 to 10 °C/s, the elongation increased by 86.5%; while with a heating rate of 1 °C/s and when the temperature increased from 800 to 900 °C, the elongation increased by 21.3%.

### 3.3. Effect of Heating Rate and Temperature on Microstructure Evolution

**Figure 5:**
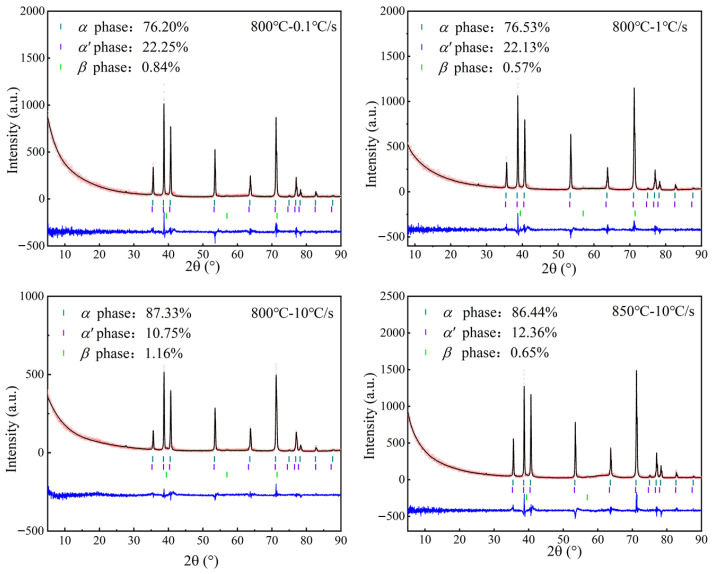
Phase volume fraction under different temperatures and heating rates.

The XRD observations of the Ti-6Al-4V under different experimental tensile temperatures and heating rates are given in Figure 5. According to the tensile test process described earlier, the high-temperature β phase completely transformed into the martensitic α’ phase during water quenching. Consequently, the high-temperature β-phase volume fraction was determined to be the sum of the α’-phase and the β-phase volume fractions under microstructure characterization [23]. At 800 °C the heating rate was augmented from 0.1 to 10 °C/s, consequently leading to a decrease in the β-phase volume fraction from 31.04% to 11.91%. This phenomenon can mainly be described as the thermodynamic non-equilibrium state of phase transformation caused by rapid heating. The alloying element V lacked sufficient time for diffusion and rearrangement, resulting in unexpected β-phase formation.

Furthermore, the tensile temperature exerts a significant influence on the phase volume fraction. As the temperature increased from 800 to 850 °C, the β-phase volume fraction ranged from 11.91% to 13.01%. This can be attributed to the effects of the β-phase stable elements, such as Mo and V, at elevated temperatures [24]. Additionally, the rate of diffusion is elevated, thereby decreasing the phase transformation time. At the same time, high-temperature deformation leads to dislocation motion and local recrystallization to release local stresses, providing nucleation positions for the β phase [25]. Therefore, increasing the temperature and decreasing the heating rate can both increase the α- to β-phase transformation volume fraction, decreasing the flow stress and increasing the elongation.

**Figure 6:**
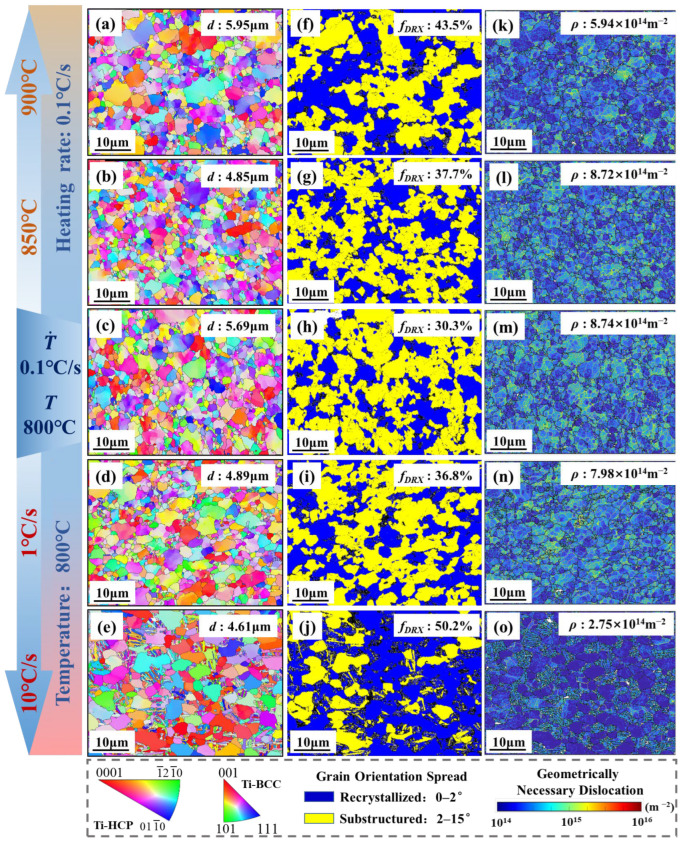
EBSD results. (**a**–**e**) IPF maps; (**f**–**j**) grain orientation spread (GOS) maps; (**h**–**o**) GND maps.

Figure 6 presents the quantified EBSD observation results, including grain size, dynamic recrystallization (DRX), and average dislocation density under different tensile temperatures and heating rates. Furthermore, the DRX fraction is represented by 0 < GOS < 2° [26]. The results show that when the heating rate increases at a temperature of 800 °C from 0.1 °C/s to 10 °C/s then the grain size decreases from 5.69 μm (Figure 6c) to 4.61 μm (Figure 6e), indicating that rapid heating inhibits grain coarsening. Meanwhile, the average dislocation density decreases from 8.74 × 10^14^ m^−2^ at a heating rate of 0.1 °C/s (Figure 6m) to 2.75 × 10^14^ m^−2^ at a heating rate of 10 °C/s (Figure 6o), and the DRX fraction increases from 30.3% at a heating rate of 0.1 °C/s (Figure 6h) to 50.2% at a heating rate of 10 °C/s (Figure 6j). This suggests that under rapid heating conditions the level of DRX fraction consumes a large number of dislocations and generates a large number of fine recrystallized grains, achieving grain refinement. This leads the specimen to have higher strength at a higher heating rate.

Temperature also has effect on the microstructure of Ti-6AL-4V. It can be observed that when the heating rate is maintained at 0.1 °C/s, the grain size decreases from 5.69 μm at 800 °C (Figure 6c) to 4.85 μm at 850 °C (Figure 6b), the DRX fraction increases from 30.3% (Figure 6h) to 37.7% (Figure 6g), and the average dislocation density decreases from 8.74 × 10^14^ m^−2^ (Figure 6m) to 8.72 × 10^14^ m^−2^ (Figure 6i). It can be seen that as the temperature increases the level of DRX fraction increases and the grains become finer. Therefore, DRX consumes a certain number of dislocations, thereby decreasing the flow stress of the tensile specimen. And high temperature causes the grains to grow at 900 °C, with the grain size increasing to 5.69 μm (Figure 6a).

## 4. Optimized Viscoplastic Constitutive Model Considering Heating Rate and VUMAT Subroutine

### 4.1. Optimized Viscoplastic Constitutive Model Considering Heating Rate

The β phase with a BCC structure has more slip systems than an α phase with HCP in Ti-6Al-4V, which has lower deformation resistance with better plasticity [27]. Therefore, the two-phases volume ratio has a great influence on the mechanical strength of the alloy. In addition, the volume ratio was found to be temperature and heating rate dependent based on experimental observations. Therefore, considering the heating rate is important for optimizing the model.

At high heating rates, the effect of heating rate on the non-equilibrium phase transformation process is defined by the following Equation (1) [28]:

(1) $$f_{T}=1-\exp-{\left[K(T-T_{0})/H\right]}^{n}$$

where $f_{T}$ is the temperature dependent transformation β-phase volume fraction, n is the exponent of the transformation from α to β, $T_{0}$ is the activation transformation temperature at the current heating rate, H is the heating rate, and K is the temperature dependent constant modeled using the Equation (2) Arrhenius equation, which is as follows:

(2) $$K\left(T\right)={\;K}_{0}\exp\left(-\frac{Q}{\mathrm{RT}}\right)$$

where $K_{0}$ is a constant and is the activation energy for diffusion from the α- to β-phase transformation. The phase transformation temperature exhibits hysteresis behavior with increasing heating rate, as shown in Equation (3) [29], which is as follows:

(3) $$T_{0\;}={\;T}_{1}\;\times \;\left[1+H/\left(a\;\times {\;H}_{max}\right)\right]$$

where $T_{1}$ is the activation temperature for the equilibrium transformation of the β phase, $H_{max}$ is the ultimate heating rate of Gleeble, and a is the material constant.

The change in the β-phase volume fraction of the Ti-6Al-4V during continuous heating is predicted by Equation (4), which is as follows, allowing the model to predict the β-phase volume fraction at different temperatures and heating rates:

(4) $$f_{\beta \;}=f_{0}+\left(1-f_{0}\right)f_{T}$$

where $f_{\beta }$, $f_{0}$, and $f_{T}$ are the β-phase volume fraction, the β phase at room temperature, and the transformation with temperature, respectively.

In this study, a viscoplastic constitutive model of Ti-6Al-4V is given to describe the evolution of dislocation density, damage, dynamic recrystallisation, and grains. This is because the difference in the crystal structure of the α phase and β phase leads to the difference in alloy properties, meaning that the viscoplastic flow of the α and β phase are modeled separately. The two-phase volume fractions are calculated by Equations (1)–(4), and the constitutive model of Ti-6Al-4V is formulated as follows [30,31]:

(5) $${\dot{\varepsilon }}_{\alpha }^{P}={\left(\frac{\sigma /(1-\omega_{D})-R_{\alpha }-k_{\alpha }}{K_{\alpha }}\right)}^{n_{1}}{\left(\bar{d}\right)}^{-\mu }$$

(6) $${\dot{\varepsilon }}_{\beta }^{P}={\left(\frac{\sigma /(1-\omega_{D})-R_{\beta }-k_{\beta }}{K_{\beta }(1-G)}\right)}^{n_{2}}$$

(7) $${\dot{\varepsilon }}^{P}={\dot{\varepsilon }}_{\alpha }^{P}\left(1-f_{\beta }\right)+{\dot{\varepsilon }}_{\beta }^{P}f_{\beta }$$

(8) $${\dot{\omega }}_{D}=\frac{\eta_{1}\sigma {\dot{\varepsilon }}^{P^{\eta_{2}}}}{{\left(1-\omega_{D}\right)}^{\eta_{3}}}$$

(9) $${\dot{\bar{\rho }}}_{\alpha }=A_{1}\left(1-{\bar{\rho }}_{\alpha }\right)\left|{\dot{\varepsilon }}_{\alpha }^{P}\right|-C_{1}{{\bar{\rho }}_{\alpha }}^{n_{3}}-\left[C_{2}{\bar{\rho }}_{\alpha }/(1-S_{\alpha })\right]{\dot{S}}_{\alpha }$$

(10) $${\dot{\bar{\rho }}}_{\beta }=A_{2}\left(1-{\bar{\rho }}_{\beta }\right)\left|{\dot{\varepsilon }}_{\beta }^{P}\right|-C_{3}{{\bar{\rho }}_{\beta }}^{n_{4}}$$

(11) $${\bar{\rho }}_{c}=q_{3}{\dot{\varepsilon }}_{\alpha }^{pq_{4}}$$

(12) $${\dot{R}}_{\alpha }=0.5B_{\alpha }{{\bar{\rho }}_{\alpha }}^{-0.5}{\bar{\rho }}_{\alpha }$$

(13) $${\dot{R}}_{\beta }=0.5B_{\beta }{{\bar{\rho }}_{\beta }}^{-0.5}{\dot{\bar{\rho }}}_{\beta }$$

(14) $${\dot{\bar{d}}}_{\alpha }=w_{1}{\bar{d}}^{{-\gamma }_{1}}+w_{2}{\dot{\varepsilon }}_{\alpha }^{P}{\bar{d}}^{{-\gamma }_{2}}-w_{3}{\dot{S}}_{\alpha }^{\gamma_{3}}(\bar{d})$$

(15) $${\dot{S}}_{\alpha }=\frac{q_{1}{\left(0.1+S_{\alpha }\right)}^{q_{2}}(1-S_{\alpha }){{\bar{\rho }}_{\alpha }}^{2}}{\bar{d}}$$

(16) $${\dot{G}}_{\alpha }=C_{G}(1-G){\dot{\varepsilon }}_{\beta }^{P}$$

(17) $$\sigma =E(1-\omega_{D})(\varepsilon^{T}-\varepsilon^{P})$$

where ${\dot{\varepsilon }}_{\alpha }^{P}$ and ${\dot{\varepsilon }}_{\beta }^{P}$ are plastic strain rates of the α and β phases, σ is effective stress, $R_{\alpha }$ and $R_{\beta }$ are corresponding hardening caused by the internal dislocation density of the α and β phases, $k_{\alpha }$ and $k_{\beta }$ are the initial yield stresses corresponding to the α and β phases, $\bar{d}$ is the normalized grain size, $\omega_{D}$ is the one-dimensional damage which represent the damage and grain size evolution in the process of thermal deformation with a value range of 0 to 1, ${\dot{\varepsilon }}^{P}$ is the total plastic strain rate, $f_{\beta }$ is the β phase volume fraction, $\rho_{\alpha i}$ and $\rho_{\beta i}$ are the volume fraction and rate of recrystallization of the *α* phase, $\rho_{\alpha }$ and $\rho_{\beta }$ are the actual dislocation densities of the α and β phases during material deformation, $S_{\alpha }$ and ${\dot{S}}_{\alpha }$ are the recrystallization volume fraction and its rate in the α phase of the material, $w_{1}$, $w_{2}$, and $w_{3}$ are material constants, G represents the spheroidization degree of the lath-like α phase, E is the Young’s modulus, $\varepsilon_{T}$ is the total strain, $\varepsilon_{P}$ is the plastic strain, $\eta_{1}$, $\eta_{2}$, $\eta_{3}$, and u are material parameters, $\gamma_{3}$ and $\gamma_{4}$ are temperature-related material parameters, and $q_{1}$ and $q_{2}$ are temperature-related constants.

The generalized following Arrhenius equation can be used to represent the temperature-dependent variables in the whole equations [32]:

(18) $$\Theta =\Theta_{0}exp\left(\frac{Q_{\Theta }}{R_{g}T}\right)$$

where Θ indicates the all the temperature dependent variables, $R_{g}$ is the ideal gas constant, and T is the temperature.

The experimental conditions of 800 °C-0.1 °C/s, 850 °C-0.1 °C/s, 900 °C-0.1 °C/s, 800 °C-1 °C/s, and 800 °C-10 °C/s were selected as the orthogonal experiments. The flow stress–strain curves and β-phase volume fractions obtained from the orthogonal experiments were used as initial values. The parameter estimation function in Matlab (R2020b) was then employed to refine the parameters in the model equation. The results of the parameter fitting are presented in Table 2.

**Figure 7:**
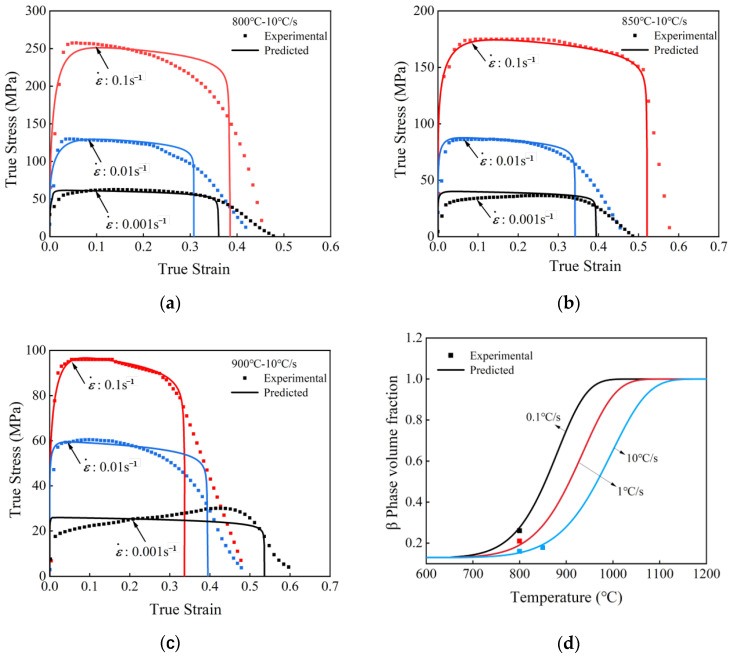
Comparison of prediction and experimental data of flow stress and phase volume fraction. (**a**) Prediction of flow stress at 800 °C–10 °C/s; (**b**) prediction of flow stress at 850 °C–10 °C/s; (**c**) prediction of flow stress at 900 °C–10 °C/s; (**d**) prediction of β-phase volume fraction.

The comparison of the stress–strain curves predicted by the model with the experimental data at heating rates of 10 °C/s, temperatures of 800 °C, 850 °C, and 900 °C, and strain rates of 0.1 s^−1^, 0.01 s^−1^, and 0.001 s^−1^ is shown from Figure 7a–c. The scattered dots represent the experimental results, and the smooth and continuous curves represent the model prediction data. The model prediction curves fit with the trend of the experimental data, thereby reflecting the phenomenon that the maximum flow stress diminishes with increasing temperature at a constant heating rate. The model demonstrates an accuracy of 92.93%. The model also performs well in predicting the phase volume fraction, as shown in Figure 7d, with an average prediction error of 5.03% for the β-phase volume fraction. The overall prediction results are reasonable, which proves the reliability of the model.

### 4.2. Current-Assisted Hot Stamping FE Model and VUMAT Subroutine

In order to apply the above established optimized viscoplastic constitutive model, an FE model was developed to simulate the current-assisted rapid heating process. The optimized viscoplastic constitutive model was transformed into VUMAT subroutines through Visual Studio 2022 software and implanted into the ABAQUS 2021 FE simulation software for the input of simulation variables.

Ti-6Al-4V sheet current-assisted rapid heating produces a non-uniform temperature field, and the faster the heating rate, the more pronounced the phenomenon of temperature non-uniformity [33]. Theoretically, if the heating time is long enough then the temperature of each component of the sheet will converge, but in practice this is not desirable. Accurate simulation of the sheet temperature field is essential for simulating the current-assisted hot stamping process, and commercially available ABAQUS 2021 software was used to build a current-assisted heating FE model. The UG 12.0 software was used to establish the shell model of the current-assisted hot stamping in accordance with the actual geometric dimensions and relative positional relationship of each component. It was imported into the FE simulation, endowed with material properties such as electrical conductivity, thermal conductivity, specific heat capacity, density, and Joule heat coefficient of the Ti-6Al-4V sheet, and the coupled thermal–electrical structural analysis was selected to solve the electric field and temperature field synchronously, The electric field load was applied to one side of the sheet to simulate the heating rate by the magnitude of the applied surface current, and the surface current was related to the heating rate. The Ti-6Al-4V sheet was divided into an 8-node reduced integral hexahedral cell thermoelectric structure mesh of 0.3 mm size, the number of integral nodes in the thickness direction was set to 5, and the hourglass control was added. The dies were set as a discrete rigid body, and the subdivision cell size was 2 mm.

**Figure 8:**
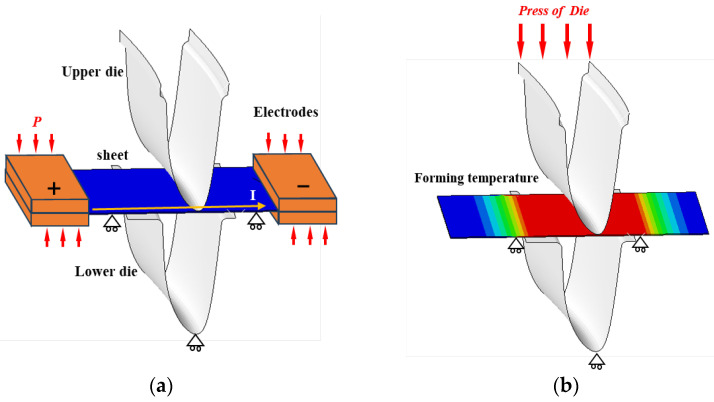
(**a**) Current-assisted rapid heating FE model; (**b**) hot stamping FE model.

To solve the problem that the finite element software cannot simulate the current-assisted heating and stamping processes simultaneously, a two-process nesting method is proposed, as shown in Figure 8. Here, process I simulates different heating rates and heats the sheet to the preset temperature; process I simulates the hot stamping process, and the number of analysis steps and incremental steps are recorded by the ODB result file calculated by the model of process I, and the predefined field of the sheet is edited by reading the ODB file to obtain the temperature information of process I; the temperature sequence from simulation process I to simulation process II is completed by reading the ODB file to set the analysis steps and incremental steps; and then the predefined field of the sheet is edited in the load of simulation process II and the temperature information of process II is obtained by reading the ODB file, and the analysis step and incremental step are set to complete the temperature sequence from simulation process II to simulation process II.

In terms of contact and boundary conditions, considering the actual stamping process, the FE model restricts the degrees of freedom of dies, and the interaction between the sheets and the die and electrode is set as a universal contact with a friction coefficient of 0.15. For the stamping process of process II, the magnitude of the pressing force is set to 0.1 MPa and the pressing speed is set to 150 mm/s.

## 5. Current-Assisted Hot Stamping of Irregular Cross-Section Components

**Figure 9:**
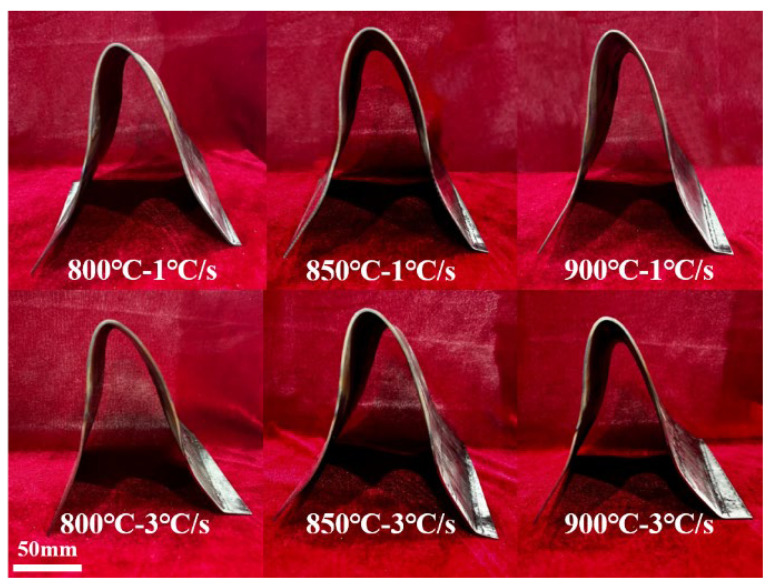
Irregular cross-section components under different stamping temperatures and heating rates.

In order to further verify the predictive ability of the optimized viscoplastic constitutive model considering heating rate in practical applications, current-assisted hot stamping forming experiments were conducted and the irregular cross-section component shown in Figure 9 was obtained. The thickness data obtained from the FE simulation were compared with the irregular cross-section components to verify the validity of the FE model. Tensile tests and microscopic tests were conducted on the irregular cross-section components to verify the predictive ability of the model.

### 5.1. Thickness of Irregular Cross-Section Components

**Figure 10:**
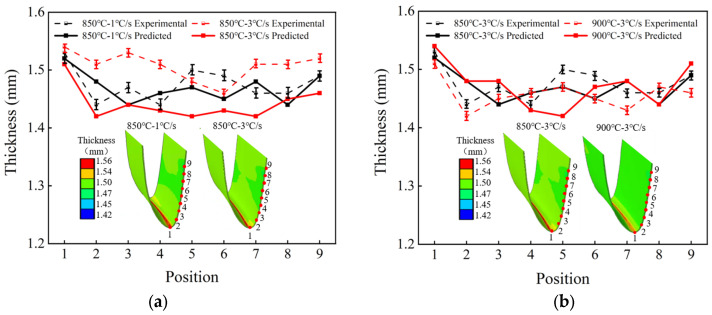
Comparison of the measured thickness of the irregular cross-section component with FE results. (**a**) Comparison of thickness at different heating rates. (**b**) Comparison of thickness at different stamping temperatures.

Figure 10 presents the cloud map of the thickness distribution of irregular cross-section components calculated from the FE model of current-assisted rapid-heating hot stamping. The irregular cross-section components show that the thickness at the bottom is thicker than that on both sides. During the stamping process of the component, the bottom area of the sheet contacted the upper die first, which led to a temperature drop, resulting in a decrease in deformability. However, the temperature loss in the suspended areas on both sides is small. Therefore, the two areas are more prone to deformation, resulting in a thinning of the thickness.

Figure 10a,b, respectively, show the comparison between the measured thickness and the FE results of the irregular cross-section components under different heating rates and different stamping temperatures. The average prediction accuracy of the FE model for thickness reached 96.96%, verifying the validity of the model.

The standard deviation was obtained through the measurement results of the thickness of the irregular cross-section components to characterize the uniformity of the thickness of the irregular cross-section components. The standard deviation of the conditions of 1 °C/s and 3 °C/s at 850 °C is 0.028 and 0.024, and when the heating rate is 3 °C/s at 900 °C the standard deviation of the thickness distribution is 0.023. Under the heating parameters of 900 °C–3 °C/s the uniformity of the thickness of the components is better.

### 5.2. Strength of Irregular Cross-Section Components

**Figure 11:**
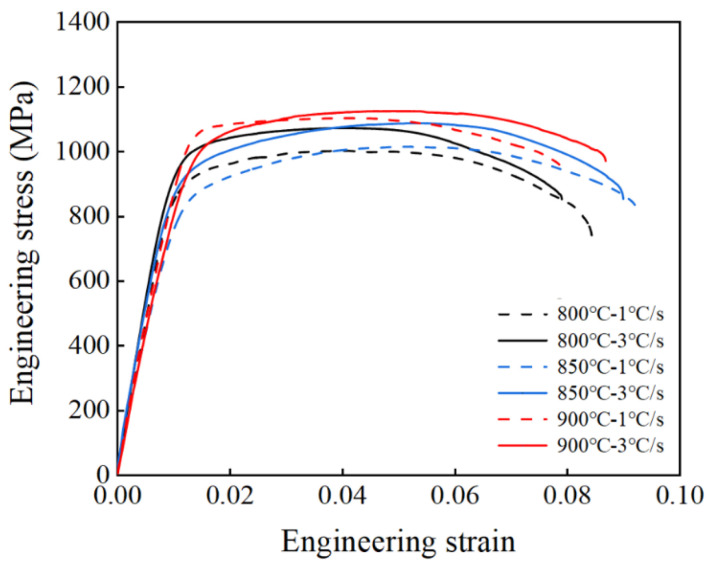
Engineering stress–strain curves of titanium alloy hot stamping irregular cross-section component with different stamping temperatures and heating rates.

Figure 11 gives the engineering stress–strain curve of the irregular cross-section component. When the heating rate is 3 °C/s the ultimate tensile strength of the irregular cross-section component increases from 1072.88 to 1125.42 MPa as the experimental temperature increases from 800 to 900 °C, and it increases from 1015.7 to 1088.39 MPa with an increase in the heating rate at 850 °C. Experiments have shown that faster heating rates and higher experimental temperatures increase the ultimate tensile strength of the components.

### 5.3. Microstructure Evolution of Hot Stamping of Irregular Cross-Section Components

**Figure 12:**
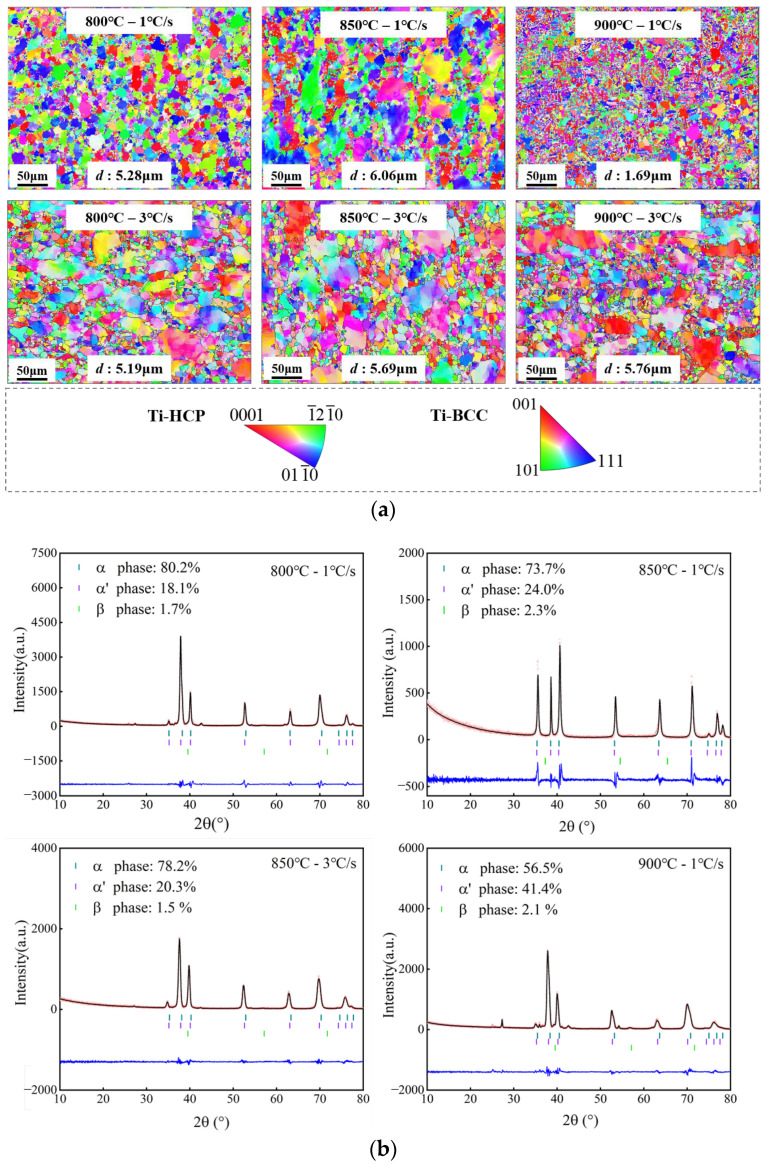
Observation results of the microstructure of irregular cross-section components. (**a**) EBSD results under different stamping temperatures and heating rates. (**b**) XRD results under different stamping temperatures and heating rates.

Figure 12a presents the EBSD quantification results of irregular cross-section components under different stamping temperatures and heating rates. Obviously, as the heating rate increases, the grain size will decrease. At 850 °C the heating rate increases from 1 to 3 °C/s and the grain size decreases from 6.06 to 5.69 μm, resulting in grain refinement, which shows that it conforms to the influence law of the above-mentioned heating rate on grain size. The phenomenon shown in the figure that the grain size of irregular cross-section components increases with the increase in stamping temperature is mainly attributed to the long processing time of the sheet, which leads to grain growth. Moreover, the sampling position is at the location during the stamping process where heat is transferred to the die and the temperature drops rapidly, resulting in insufficient dynamic recrystallization and thus coarse grains.

The XRD test results of the irregular cross-section components are shown in Figure 12b. As the experimental temperature increased from 800 to 900 °C, the β-phase volume fraction increased from 19.8% to 43.5% at the heating rate of 1 °C/s. As the heating rate increased from 1 to 3 °C/s, the β-phase volume fraction decreased from 26.3% to 21.8% at an experimental temperature of 850 °C. From the perspective of the microstructure, the change in the β-phase volume fraction is consistent with the previous experimental results, which verifies the effect of the heating rate.

The phenomenon of grain refinement in the irregular cross-section component at 900 °C with a heating rate of 1 °C/s in the figure is mainly attributed to the slow heating rate and the high target temperature, resulting in a long holding time at high temperature and complete dynamic recrystallization, which refines the grains. It can also be observed from Figure 12b that the β-phase volume fraction under this condition is 43.5%. During the cooling process of the irregular cross-section component martensitic transformation occurs, generating a dense and randomly distributed α’ phase. This confirms the superior macroscopic mechanical properties of the irregular cross-section component under the condition of 900 °C-1 °C/s.

**Figure 13:**
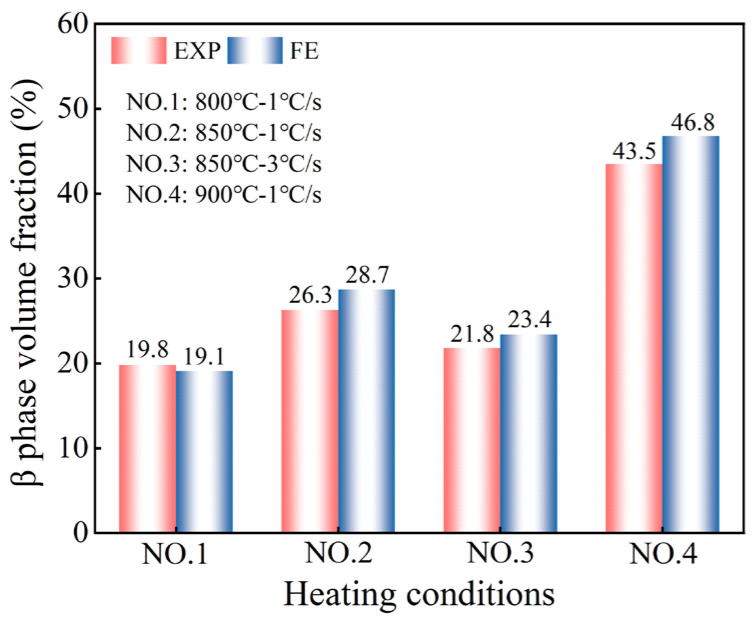
The comparison of β-phase volume fraction predictions with experimental results.

The comparison of phase volume fraction predictions with experimental results is given in Figure 13. All the conditions which were obtained for the tests agreed, with an average prediction error of 7.24% for the β-phase volume fraction, further demonstrating the effectiveness of the model in calculating microstructures for the hot stamping process considering the heating rate.

## 6. Conclusions

In this study, through hot tensile tests, model optimization, and current-assisted hot stamping experiments and FE simulations, the hot flow behavior, grain size, and phase evolution and formability under various heating rates and temperatures of Ti-6Al-4V were investigated deeply. The results provide effective guidance for the formulation and optimization of the current-assisted hot stamping process of Ti-6Al-4V components. The following conclusions are drawn:

(1) For the evolution of mechanical properties under different heating conditions, the maximum flow stress decreased with an increase in the tensile temperature range between 850 and 950 °C, while the elongation showed an opposite trend. With the heating rate increasing from 1 to 10 °C/s both the maximum flow stress and elongation generally increased.
(2) For the evolution of the microstructure under different heating conditions, the β-phase volume fraction increases with a decrease in the heating rate at the same temperature. Rapid heating promotes dynamic recrystallization, effectively decreasing dislocation density and inhibiting grain growth.
(3) The optimized viscoplastic constitutive model has successfully predicted both the flow stress and phase transformation in Ti-6Al-4V with different heating conditions. The prediction accuracies for flow stress and β-phase volume fraction are 92.93% and 94.97%, respectively.
(4) A current-assisted hot stamping FE model was established with a VUMAT subroutine developed based on the optimized viscoplastic constitutive model. The non-uniform temperature field of the current-assisted heating sheet and the inheritance of the temperature field during the heating and stamping processes were successfully simulated. In addition, the simulated thickness and β-phase volume fraction agreed well with the stamping experimental results, with accuracies of 96.96% and 92.76%.

## Figures and Tables

**Table 1:** Main chemical composition of Ti-6Al-4V (wt%) [20].

Al	V	Fe	H	C	N	O	Ti
6.10	4.20	0.15	0.007	<0.01	<0.01	0.13	Remain

**Table 2:** Constants of viscoplastic constitutive model.

Parameter	Value	Parameter	Value	Parameter	Value	Parameter	Value
n (–)	0.4	$T_{1}$ (°C)	650	$H_{max}$ (°C)	1000	$f_{0}$ (%)	0.13
Q (J/mol)	4450	R [J/(mol·K)]	8.314	a (–)	0.4	$K_{0}$ (–)	4.52 × 10^16^
$k_{\alpha 0}$ (MPa)	2.133 × 10^3^	${Qk}_{\alpha 0}$ (J/mol)	110,100	$q_{30}$ (–)	0.02875	${Qq}_{30}$ (J/mol)	18,950
$k_{\beta 0}$ (MPa)	6.959	${Qk}_{\beta 0}$ (J/mol)	31,920	$q_{40}$ (–)	0.00724	${Qq}_{40}$ (J/mol)	34,650
$K_{\alpha 0}$ (MPa)	0.7454	${QK}_{\alpha 0}$ (J/mol)	61,000	$\omega_{10}$ (–)	8.4 × 10^−10^	${Q\omega }_{10}$ (J/mol)	0.1622
$K_{\beta 0}$ (MPa)	70,440	${QK}_{\beta 0}$ (J/mol)	−56,180	$\omega_{20}$ (–)	9.6 × 10^−10^	${Q\omega }_{20}$ (J/mol)	0.602
$n_{10}$ (–)	1.256	${Qn}_{10}$ (J/mol)	3894	$\omega_{30}$ (–)	10.75	${Q\omega }_{30}$ (J/mol)	0.4505
$n_{20}$ (–)	0.1589	${Qn}_{20}$ (J/mol)	9491	$\gamma_{10}$ (–)	1.931	${Q\gamma }_{10}$ (J/mol)	5804
$n_{30}$ (–)	6.451 × 10^−4^	${Qn}_{30}$ (J/mol)	76,060	$\gamma_{20}$ (–)	3.286	${Q\gamma }_{20}$ (J/mol)	4098
$n_{40}$ (–)	9.269 × 10^−4^	${Qn}_{40}$ (J/mol)	68,580	$\gamma_{30}$ (–)	0.8447	${Q\gamma }_{30}$ (J/mol)	3427
$A_{10}$ (–)	11.24	${QA}_{10}$ (J/mol)	19,340	$\gamma_{40}$ (–)	3.346	${Q\gamma }_{40}$ (J/mol)	6331
$A_{20}$ (–)	13.22	${QA}_{20}$ (J/mol)	−1166	$\eta_{10}$ (–)	0.1555	${Q\eta }_{10}$ (J/mol)	70.93
$C_{10}$ (s^−1^)	0.0076	${QC}_{10}$ (J/mol)	30,480	$\eta_{20}$ (–)	1.838	${Q\eta }_{20}$ (J/mol)	−5602
$C_{20}$ (s^−1^)	5.843	${QC}_{20}$ (J/mol)	10,990	$\eta_{30}$ (–)	1840	${Q\eta }_{30}$ (J/mol)	−45,500
$C_{30}$ (s^−1^)	0.00303	${QC}_{30}$ (J/mol)	51,820	$E_{0}$ (MPa)	890	${QE}_{0}$ (J/mol)	0
$q_{10}$ (–)	7.974 × 10^−2^	${Qq}_{10}$ (J/mol)	30.07	$B_{\alpha 0}$ (MPa)	3.99 × 10^−4^	${QB}_{\alpha 0}$ (J/mol)	55,900
$q_{20}$ (–)	3.59 × 10^−3^	${Qq}_{20}$ (J/mol)	68,870	$B_{\beta 0}$ (MPa)	45.52	${QB}_{\beta 0}$ (J/mol)	12,280

## Data Availability

The original contributions presented in this study are included in the article. Further inquiries can be directed to the corresponding author.
